# High Mobility Group Box-1: A Missing Link between Diabetes and Its Complications

**DOI:** 10.1155/2016/3896147

**Published:** 2016-10-25

**Authors:** Han Wu, Zheng Chen, Jun Xie, Li-Na Kang, Lian Wang, Biao Xu

**Affiliations:** Department of Cardiology, Drum Tower Hospital, Nanjing University Medical School, Nanjing 210008, China

## Abstract

High mobility group box-1 (HMGB-1), a damage-associated molecular pattern, can be actively or passively released from various cells under different conditions and plays a pivotal role in the pathogenesis of inflammation and angiogenesis-dependent diseases. More and more evidence suggests that inflammation, in addition to its role in progression of diabetes, also promotes initiation and development of diabetic complications. In this review, we focus on the role of HMGB-1 in diabetes-related complications and the therapeutic strategies targeting HMGB-1 in diabetic complications.

## 1. Introduction

Diabetes is evolving as an epidemic disease, which affects about 400 million people worldwide. It is a metabolic disorder characterized by hyperglycemia due to defective insulin secretion or insulin resistance. Growing evidence on the involvement of inflammation in diabetes has made it an attractive topic. HMGB-1, an inflammatory mediator, can be released from various cells under different stress conditions. Over the past decade, a great gain of information about HMGB-1 in inflammatory diseases has been made. This review will give an overview of recent advances in HMGB-1 in diabetes, diabetic cardiovascular complications, diabetic nephropathy (DN), and diabetic retinopathy (DR), and then we will focus on therapeutic strategies targeting HMGB-1.

## 2. Introduction of HMGB-1

We will first introduce some definitions that will be used throughout the manuscript. These include ROS (reactive oxygen species), RAGE (receptor for advanced glycation end product), TLR2 (Toll-like receptor-2), TLR4 (Toll-like receptor-4), NF-*κ*B (nuclear factor-*κ*B), MyD88 (myeloid differentiation factor-88), p38MAPK (p38 mitogen-activated protein kinase), ERK (extracellular signal-regulated kinase), and JNK (c-Jun N-terminal kinase).

**Figure 1:**
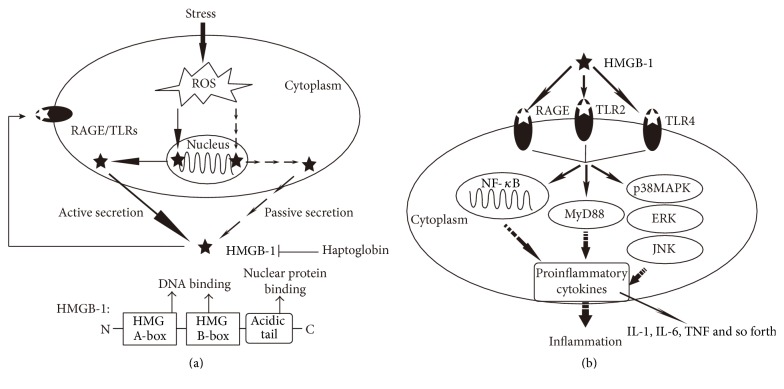
Schema depicting the structure of HMGB-1 and the molecular mechanisms responsible for the role of HMGB-1 in inflammation. (a) HMGB-1 translocates from the nucleus to the cytoplasm under oxidative stress condition and then is actively (immune or active inflammatory cells) or passively (death, apoptosis, or necrosis cells) released outside the cells. Once it is released into the extracellular space, HMGB-1 in turn promotes oxidative stress by binding to its receptors (such as RAGE, TLR2, and TLR4). (b) Extracellular HMGB-1 binds to its receptors and induces inflammatory response via various signaling pathways involving NF-*κ*B, MyD88, p38MAPK, ERK, and JNK.

HMGB-1, a member of HMG protein superfamily, was first named 40 years ago because of its high mobility in polyacrylamide gel electrophoresis system [1, 2]. Human HMGB-1 is an about 30 kDa protein consisting of 215 amino acids and includes three distinct domains: two positive charged domains (A-box and B-box) composed of about 80 amino acids separately and a negatively charged acidic C-terminal tail composed of 30 amino acids. Furthermore, another 24 amino acids are used to link the domains [3]. HMG boxes A and B are able to mediate DNA binding, while the acidic C-tail can regulate the affinity of binding to distorted DNA structures via binding to other nuclear proteins [4] (Figure 1).

HMGB-1 is a ubiquitous nuclear protein for maintaining DNA structure in various cells, but it can be actively or passively released under stress conditions. For example, in inflammation-associated immune cells such as monocytes and macrophages, HGMB-1 translocates from nuclear to special cytoplasmic organelles and is actively released outside the cells under stress. Besides, HMGB-1 may fail to bind to chromatin and is passively released into the extracellular space from cells undergoing necrosis, apoptosis, and injury [5]. However, secretion of HMGB-1 was recently shown to be negatively regulated by haptoglobin. Yang et al. found that haptoglobin-HMGB-1 complexes elicited anti-inflammatory cytokines in macrophages, suggesting a mechanism for haptoglobin modulation of HMGB-1 action [6] (Figure 1(a)).

Once it is released outside the cells, extracellular HMGB-1 functions as a multifunctional cytokine in many pathophysiological processes. There are several receptors for HGMB-1, but of importance are receptor for advanced glycation end product (RAGE), Toll-like receptor-2 (TLR2), and Toll-like receptor-4 (TLR4) [7]. By binding to its receptors, HMGB-1 induces nuclear translocation of activated nuclear factor-*κ*B (NF-*κ*B), leading to secretion of proinflammatory cytokines including tumor necrosis factor-*α* (TNF-*α*), interleukin-6 (IL-6), and interleukin-1 (IL-1) [8]. In addition, extracellular signal-regulated kinase (ERK), p38 mitogen-activated protein kinase (p38MAPK), c-Jun N-terminal kinase (JNK), and myeloid differentiation factor-88 (MyD88) are also involved in HMGB-1-mediated signaling pathways [9–11], which finally contributes to immunoinflammatory response via proinflammatory cytokine secretion. It is worth mentioning that proinflammatory cytokines in turn promote more leakage of HMGB-1 from cells [12, 13], suggesting that HMGB-1-mediated signaling pathways are amplified by a positive feedback loop involving inflammation (Figure 1(b)).

## 3. HMGB-1 and Diabetes

In diabetic condition, HG or AGEs can induce HMGB-1 resection via oxidative stress. HMGB-1-RAGE interaction promotes islet cell apoptosis in diabetes by inducing oxidative stress, contributing to insulin deficiency. In addition, released HMGB-1 induces JNK/p38MAPK activation via binding to TLR4, which leads to insulin resistance via inhibitory phosphorylation of insulin receptor substrate. Finally, HMGB-1-induced insulin deficiency and insulin resistance contribute to the development of diabetes.

Additional definitions include HG (high glucose), AGEs (advanced glycation end products), ROS (reactive oxygen species), RAGE (receptor for advanced glycation end product), TLR4 (Toll-like receptor-4), JNK (c-Jun N-terminal kinase), p38MAPK (p38 mitogen-activated protein kinase), and IRS (insulin receptor substrate).

Diabetes has been widely recognized as a modern-day disease with a significant increase in morbidity and mortality worldwide. It is characterized as a chronic inflammation condition, and inflammatory mediators such as IL-1beta, TNF-*α*, and IL-6 are increased in diabetes.

More and more evidence shows upregulation of IL-1beta in serum and pancreatic islets from diabetes [14, 15]; furthermore, genetic or pharmacologic inhibition of IL-1beta system has been shown to improve beta-cell function, insulin secretion, and glycemic control in proof-of-concept clinical studies and animal experiments [16–19]. Thus, the role of inflammation in diabetes has long been appreciated and gained broad attention [20].

It is demonstrated that subjects with diabetes exhibited higher serum HMGB-1 levels compared with nondiabetic patients [21]. Since then, the role of HMGB-1 in diabetes has been confirmed by other studies [22–24]. HMGB-1 was shown to be increased at the onset of cystic fibrosis-related diabetes [25] and was associated with indexes of glucose metabolism and body mass index in diabetes [24, 25]; however, treatment with insulin lowered its levels induced by glucose infusion [25, 26]. In addition, our recent work has also provided convincing evidence that type 1 diabetic mice exhibited markedly elevated serum HMGB-1 concentration compared with the controls [27].

**Figure 2:**
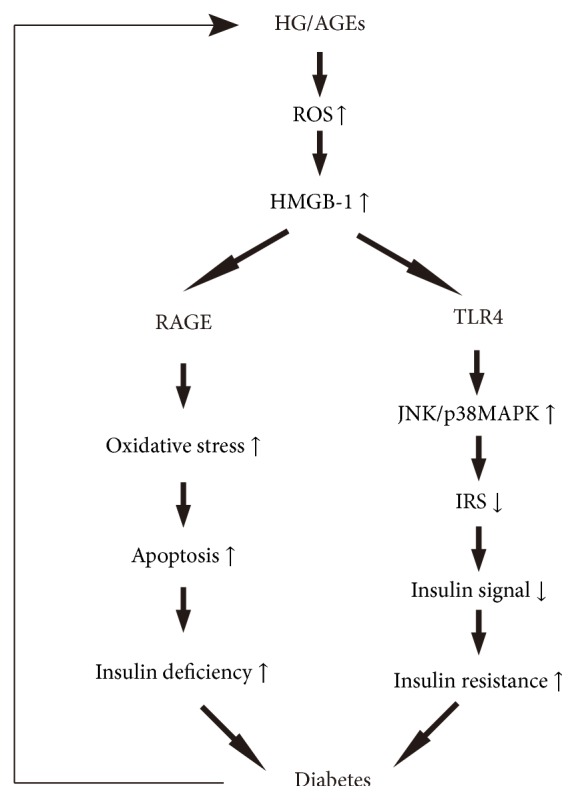
Schematic representation of HMGB-1-mediated cellular signaling pathways in islet cells.

Furthermore, more and more in vitro experiments showed increased HMGB-1 expression in various cells upon exposure to high glucose [11, 28–30]. Wang et al. [30] demonstrated that high glucose induced HMGB-1 translocation through NADPH oxidase and PKC-dependent way in vascular smooth muscle cells. In diabetes, the elevated level of glucose promotes glycation of plasm proteins through a nonenzymatic process, which leads to advanced glycation end products (AGEs). AGEs contributed to metabolic memory effect as they exhibited their harmful effects for a long time via binding to RAGE even if the glucose was controlled well in diabetes. Recently, we found that HMGB-1 was upregulated in AGEs-induced endothelial progenitor cells (EPCs) via increased oxidative stress [27]. In line with our findings, another experiment showed that high-glucose-induced HMGB-1 expression in tubular epithelial cells was reduced by N-acetylcysteine (NAC), an antioxidant [11], suggesting that high glucose may lead to HMGB-1 expression via oxidative stress (Figure 2).

More and more studies showed a link between AGEs and HMGB-1 [31–33]. In vitro experiment has shown that AGEs induced endothelial dysfunction via HMGB-1-mediated inflammation and oxidative stress in human umbilical vein endothelial cells [34]. AGEs induced translocation and release of HMGB-1 from tubular epithelial cells; furthermore, HMGB-1 knockdown inhibited AGEs-induced expressions of cytokines, suggesting that HMGB-1 enhanced AGEs-induced expressions of cytokines. In addition, the effect of AGEs and HMGB-1 in epithelial cells was dependent on RAGE-mediated signaling pathway [35]. Thus, RAGE transduced the signals of AGEs and HMGB-1, and HMGB-1 amplified AGEs-mediated signaling pathways by binding to RAGE [36].

Islet beta-cell dysfunction and insulin resistance are two important pathogeneses of DM. Lee et al. [37] investigated the expression of RAGE in islet cells and the effect of HMGB-1 on islet cells. They surmised that HMGB-1-RAGE interaction contributed to islet cell apoptosis in type 2 diabetes by inducing oxidative stress. Activation of TLR4, another receptor of HMGB-1, activated proinflammatory kinases (such as p38MAPK and JNK) that impaired insulin signal transduction via inhibitory phosphorylation of insulin receptor substrate [38], suggesting the involvement of HMGB-1 in insulin resistance. In addition, pharmacological inhibition of TLR4 protected insulin resistance in fat-induced rats [39]. However, in contrast to these findings, another report showed that HMGB-1 promoted insulin secretion in islet beta-cells [40]. The discrepancy between the two results may be related to the cell lines and the exposure times to HMGB-1 used in the experiments. It is crucial to note that HMGB-1 may induce islet cells apoptosis and insulin resistance via binding to TLR4 and then contribute to initiation and development of DM (Figure 2).

Taken together, not only is HMGB-1 released in response to hyperglycemia via oxidative stress in diabetes, but also it contributes to the progression of diabetes via inducing islet cells apoptosis and insulin resistance, though the underlying mechanisms are not clearly understood.

## 4. HMGB-1 and Cardiovascular Complications

Cardiovascular complications including coronary artery disease and diabetic cardiomyopathy have become the leading causes of morbidity and mortality in diabetes. A growing body of evidence has indicated the important role of HMGB-1 in plaque formation, rupture, and thrombosis, which are all pathogenic phenomenons in coronary artery disease. In addition, clinical studies showed elevated circulating HMGB-1 in coronary artery disease [21, 64, 65]. Higher level of HMGB-1 was reported to be associated with the severity of coronary artery stenosis and it may be a predictor for coronary atherosclerosis in young patients with chest pain and for cardiovascular mortality in acute coronary syndrome [64, 66–68]. As described in Table 1, increasing evidence has summarized the contribution of HMGB-1 to coronary artery disease with diabetes.

Two clinical studies showed significantly higher serum HMGB-1 level in coronary artery disease patients with diabetes than in patients without diabetes [21, 41]. Furthermore, a recent study reported that serum HMGB-1 was positively related to HbA1c level and was an independent predictor for coronary artery disease patients with diabetes [41].

Our recent study indicated that HMGB-1 could amplify oxidative stress in AGEs-induced endothelial progenitor cells, a subtype of CD34-positive cells [27]. In line with our studies, Feng and colleagues indicated that the interaction of HMGB-1 and its receptors triggered the oxidative stress in endothelial dysfunction [34]; similarly, serum HMGB-1 concentration was shown to reflect endothelial dysfunction in diabetes [22]. Recently, the increased HMGB-1 expression was found to be positively correlated with arterial stenosis, oxidative stress, and inflammation in diabetic patients with peripheral arterial occlusive disease [46]. In vitro experiment showed that microRNA-24 attenuated high-glucose-induced vascular smooth muscle cell proliferation and migration by targeting HMGB-1. Furthermore, downregulation of HMGB-1 inhibited production of TNF-*α* and IL-6 via inhibition of NF-*κ*B [52]. Yamashita and coworkers demonstrated that there were fewer CD34-positive cells and increased expression of HMGB-1 in acute coronary thrombi with DM [42]. In a thrombin-induced disseminated intravascular coagulation (DIC) rat model, HMGB-1 was shown to promote development of thrombosis [69]. The above evidence suggested that elevated HMGB-1 in diabetes induced oxidative stress, impaired endothelial repair, caused dysfunction of vascular smooth muscle and thrombosis, and finally led to ischemic event associated with diabetes.

Diabetic cardiomyopathy, a new diabetic complication independent of coronary artery disease and hypertension, has been increasingly recognized in diabetic patients by clinicians. More and more investigations indicated that HMGB-1 was diffusely expressed in myocardium of diabetic mice and in hyperglycemia-induced cardiomyocytes [28, 43, 44]. Wang et al. [44] found that high glucose could induce HMGB-1 expression, translocation, and secretion in cardiomyocytes and fibroblasts. Similarly, HMGB-1 was reported to be upregulated in fibroblasts, macrophages, and cardiomyocytes isolated from diabetes mellitus subjects [47]. Besides that, a clinical study showed increased serum HMGB-1 in heart failure patients which was related to the severity of heart failure in diabetic patients [70]. Thus, there is a close link between HMGB-1 and heart failure. Wang and coworkers [44] reported that genetic inhibition of HMGB-1 improved myocardial function in diabetic cardiomyopathy, suggesting that HMGB-1 contributes to myocardial dysfunction in diabetes and inhibition of HMGB-1 might have therapeutic potential in treatment of diabetic cardiomyopathy.

Mechanistically, complex and highly diversified mechanisms are involved in the pathogenesis of diabetic cardiomyopathy, such as oxidative stress, inflammation, myocardial fibrosis, and apoptosis.

In a mice model of diabetic cardiomyopathy, HMGB-1 inhibition ameliorated left ventricular remodeling. In vitro, incubation of high glucose or HGMB-1 induced expression of collagens and profibrogenic factors, which were reversed by inhibition of HGMB-1 [44]. Recently, similar studies showed that HMGB-1 released from necrotic myocardial cells or active fibroblasts could induce fibroblast activation in vitro as well as myocardial fibrosis in vivo through TLR4-dependent signaling and ERK-dependent signaling pathway [71, 72].

Inflammation, another pathologic process in diabetic cardiomyopathy, was confirmed by increased expression of HMGB-1 in the ventricular myocardium as well as increased inflammatory cell infiltration and TNF-*α* expression. Interestingly, in vitro and in vivo experiments showed that hyperglycemia-induced HMGB-1 was reversed by treatment of resveratrol or metformin, both of which were considered as antioxidants [43, 45]. In addition, cultured cardiomyocytes showed increased cell death and HGMB-1 expression in response to H_2_O_2_ treatment [73]. These data indicated that the cross talk between oxidative stress and HMGB-1-mediated signaling pathway played an important role in diabetic cardiomyopathy.

## 5. HMGB-1 and DN (Diabetic Nephropathy)

DN has become one of the most devastating diabetic complications, the leading cause of end stage renal disease. HMGB-1 has been suggested to participate in inflammatory progress, contributing to the development and progression of DN (Table 2).

An increase of inflammatory factors including HMGB-1 was reported in DN [49, 74, 75]. In vitro, high glucose induced HMGB-1 and proinflammatory cytokines in renal mesangial cells and proximal tubular epithelial cells; furthermore, NF-*κ*B mediated high-glucose-induced promotion of proinflammatory cytokines, suggesting that high glucose may induce HMGB-1 expression as well as inflammatory cytokines via activation of NF-*κ*B signaling pathway [11, 48, 51].

One of the earliest investigations on the role of HMGB-1 in DN was prompted by the notion that HMGB-1 was a ligand of RAGE and TLRs in DN [49, 76]. In addition, upregulated HMGB-1 along with increased expressions of TLR2 and TLR4 in tubules was investigated in renal sections from DN patients [48, 49]. Indeed, the results were confirmed by in vitro studies that high glucose induced expressions of TLR2 and TLR4 and their downstream proteins in human proximal tubular epithelial cells, renal tubular epithelial cells, and podocytes [11, 48–50]. However, inhibition of TLR2 or TLR4 in diabetes exhibited reduced inflammatory responses [48, 50, 77], indicating that TLR2 or TLR4 might mediate HMGB-1-induced inflammation in DN.

In addition to inflammation, HMGB-1 and its receptors were also involved in fibrotic process of DN [35, 50, 77]. It has been established that RAGE activation promoted inflammation and fibrosis in DN [75, 78, 79]. Recently, an in vitro study showed that HMGB-1 enhanced the AGEs-induced expression of profibrogenic factors in renal tubular epithelial cells via RAGE-dependent signaling, suggesting that HMGB-1 may play an important role in the renal fibrotic process of DN via binding to RAGE [35]. Furthermore, elevated HMGB-1 along with increased interstitial fibrosis was reported in streptozotocin-induced DN; in contrast, marked reductions in interstitial fibrosis were evident in TLR4-deficient mice [50, 77]. Moreover, DN-induced MyD88 expression and NF-*κ*B activity were significantly reversed in global TLR4-knockout mice [77]. An in vitro study showed that high glucose directly promoted TLR4 activation in podocytes and tubular epithelial cells and then resulted in NF-*κ*B activation [50].

Thus, HMGB-1 may promote fibrosis and inflammation in diabetic kidney via its receptor including RAGE, TLR2, and TLR4; in addition, MyD88 and NF-*κ*B may also be involved in this process.

## 6. HMGB-1 and DR (Diabetic Retinopathy)

DR is one of the most important complications of DM and is the leading cause of preventable blindness in working-age adults. From the first study demonstrating higher level of HMGB-1 in the vitreous from patients with DR [57] to recent work in a DR rat model suggesting that HMGB-1 expression in diabetic retina was significantly higher than in the control [62], the evidence points strongly to the role of HMGB-1 in different pathological processes of DR (Table 3).

Increasing evidence indicates that oxidative stress is a central regulator of HMGB-1's activity in inflammation and cell death [61, 80]. In rat retinal ganglion cell line-5 (RGC-5 cell), AGEs stimulated release of HMGB-1 in cell supernatant as well as a significant increase of intracellular reactive oxygen species (ROS) production; however, NAC blocked HMGB-1 production via inhibition of oxidative stress [56]. Ethyl pyruvate, a well-known antioxidant substance, was shown to inhibit the upregulation of HMGB-1 in the retinas of oxygen-induced retinopathy [81]. The results indicated that HMGB-1 promoted development of DR via oxidative stress.

Inflammation has been considered as a contributing factor to the development of DR. HMGB-1, an alarm of inflammation, has also been reported to be upregulated in epiretinal membranes and vitreous fluid from DR [57, 58]. In vitro, AGEs or high glucose induced significant release of HMGB-1 from RGC-5 cells [56, 60]. Recently, cytoplasmic translocation of HMGB-1 was found in diabetes and high-glucose-induced retinal pericytes, which was dependent on RAGE/NF-*κ*B pathway [59]. In a clinical study, levels of HMGB-1 were positively correlated with monocyte chemoattractant protein-1 (MCP-1) and soluble intercellular adhesion molecule-1 (sICAM-1) in the vitreous fluid from patients with PDR, suggesting the role of HMGB-1 in inflammation of PDR [57].

HMGB-1 signaling pathway components including receptors for HMGB-1 and NF-*κ*B were significantly upregulated in type 2 diabetic retinas and in high-glucose-treated retinal cells (acute retinal pigment epithelitis-19 cells (ARPE-19 cells) and RGC-5 cells) [29, 60]. Intravitreal administration of HMGB-1 or high-glucose-treated ARPE-19 cells induced significant upregulation of inflammatory signaling molecules, which was attenuated by inhibition of HMGB-1 [29, 58]. Furthermore, inhibition of HMGB-1 decreased expressions of TLR4 and NF-*κ*B in high-glucose-induced RGC-5 cells [60]. These data suggested that diabetes-induced HMGB-1 possibly interacted with RAGE/TLR4 and activated ERK and NF-*κ*B to generate an inflammatory response in DR.

In clinical investigations, the mean levels of HMGB-1 and angiogenesis biomarkers were significantly higher in PDR patients than in nondiabetic patients; furthermore, there was a positive correlation between vitreous fluid levels of HMGB-1 and sVE-cadherin, an angiogenesis biomarker [53, 55]. Incubation of human retinal microvascular endothelial cell (HRMEC) with HMGB-1 and intravitreal injection of HMGB-1 significantly increased expression of VEGF and VEGFR, both of which were shown to promote angiogenesis [54, 82]. In addition, AGEs-induced VEGF-A production in RGC-5 cells was suppressed by glycyrrhizin, an inhibitor of HMGB-1, suggesting that HMGB-1 was implicated in the angiogenesis of DR via regulating VEGF-A production [56, 82]. In line with the findings, a recent study demonstrated that HMGB-1 promoted lymphangiogenesis via TLR4/NF-*Κ*B/MMP9 signaling pathway, indicating that HMGB-1 may play an important role in diabetic retinopathy by modulating MMP9 [83].

Besides inflammation and angiogenesis, vascular permeability and neurodegeneration are also two important pathogeneses of DR.

HMGB-1 reduced transendothelial electrical resistance of retinal endothelial cells in vitro and intravitreal administration of HMGB-1 to normal rats induced significantly increased retinal vascular permeability. In addition, diabetes induced upregulation of RAGE, ERK, and NF-*κ*B; however, oral administration of glycyrrhizin, a specific inhibitor of HMGB-1, attenuated NF-*κ*B activation. The results showed that HMGB-1 induced vascular barrier dysfunction via HMGB-1/RAGE/NF-*κ*B signaling pathway [58].

Inverse correlation between serum levels of brain-derived neurotrophic factor (BDNF) and HMGB-1 was observed in proliferative diabetic retinopathy patients, and intravitreal administration of HMGB-1 induced decreased BDNF in rat retinas, suggesting that HGMB-1-induced downregulation of BDNF might be a therapeutic target to prevent DR neurodegeneration [61, 63]. Besides, HMGB-1 was found to promote neuropathy via regulating MMP9 [84, 85], which reveals a possible role for HMGB-1/MMP9 pathway in neurodegeneration during DR.

## 7. Therapeutic Strategies Targeting HMGB-1 in Diabetic Complications

Since HMGB-1 was involved in diabetes, some HMGB-1-targeted therapeutic strategies were found to intervene in diabetic complications.

Several hypoglycemic drugs have been considered as HMGB-1-targeted drugs in diabetes care. It has been demonstrated that insulin infusion suppressed HMGB-1/TLRs in monocytes of type 1 diabetes patients [26]. An in vitro experiment showed that metformin protected hyperglycemia-induced cardiomyocytes injury via inhibition of HMGB-1/RAGE expression [45].

Glycyrrhizin is a natural triterpene isolated in licorice and has been widely shown to protect against diabetic complications. Oral administration of glycyrrhizin protected retinal vascular barrier via inhibition of HMGB-1-mediated inflammatory signaling pathway in DR [58]. In addition, constant glycyrrhizin intake from the onset of diabetes significantly attenuated HMGB-1 expression and activated ERK1/2 in retina [86]. We previously found that glycyrrhizin inhibited HMGB-1 expression in AGEs-induced EPCs, indicating the possible effect of glycyrrhizin in EPCs dysfunction in diabetes [27].

As mentioned above, oxidative stress was considered as an important player in regulating HMGB-1 in diabetes. Thus, antioxidant agents were shown to prevent diabetic complications via inhibition of HMGB-1. In diabetic mice, treatment with superoxide dismutase mimetic Mn(III)tetrakis(4-benzoic acid) porphyrin chloride (MnTBAP) normalized expression of HMGB-1/RAGE pathway [31]. Resveratrol, an antioxidant compound in red wine and vegetable foods, has been shown to prevent morphofunctional ventricular remodeling and attenuated HMGB-1 expression in type 1 diabetic rats [43]. Recently, we demonstrated that AGEs-induced HMGB-1 expression was attenuated by treatment with NAC, a potent antioxidant [27].

Astilbin, a flavonoid compound found in* Smilax china* L., has been shown to protect against ischemia-reperfusion injury in diabetic rat heart via inhibition of HMGB-1/NF-*κ*B signaling pathway [87]. Besides, several other agents such as ethyl pyruvate [81], quercetin [88–90], atorvastatin [91], and simvastatin [92] were explored in various diseases by targeting HMGB-1; however, their protection effects in diabetes and its complications needed further investigations.

## 8. Conclusions

Diabetes induces HMGB-1 expression in various cells via oxidative stress; however, elevated HMGB-1 promoted initiation and development of diabetes via induction of insulin deficiency and insulin resistance. Furthermore, HMGB-1 mediated diabetic complications including CAD, DCM, DN, and DR via various signaling pathways. Though several drugs were shown to prevent diabetic complications via targeting HMGB-1, much further research is needed to explore novel HMGB-1-targeted therapeutics in diabetes care.

## Figures and Tables

**Table 1:** Functions of HMGB-1 in diabetic cardiovascular complications.

Diseases/pathological phenomena	Involved signal molecule(s)	Notes	Ref.
CAD	HMGB-1	Elevated serum levels in diabetic patients with CAD.	[21]

CAD	HMGB-1	Serum HMGB-1 was increased in CAD patients with T2DM.	[41]

AMI	HMGB-1	HMGB-1 expression in thrombus was higher in AMI patients with DM and was positively correlated with blood glucose.	[42]

DCM	HMGB-1	Increased HMGB-1 expression in myocardial tissue of DCM.	[43]

DCM	HMGB-1, TGF-1beta, collagens, MMP2, MMP9, ERK, JNK, Akt	HMGB-1 promoted myocardial fibrosis and dysfunction in DCM. HMGB-1 mediated HG-induced TGF-1beta, collagens, and MMPs expressions in cardiac cells. Involvement of HMGB-1 in HG-induced cardiac fibroblasts proliferation and migration.	[44]

DCM	HMGB-1, RAGE	HMGB-1/RAGE may be involved in HG-induced cardiomyocytes injury.	[45]

Foot atherogenesis	HMGB-1, VCAM, NF-*κ*B	HMGB-1-induced inflammation mediated pathogenesis of diabetic foot atherogenesis.	[46]

I/R injury	HMGB-1, RAGE, NF-*κ*B, TNF-*α*, IL-6	HG induced inflammatory response via HMGB-1/RAGE/NF-*κ*B pathway in I/R models.	[47]

Endothelial dysfunction	HMGB-1, ROS	HMGB-1 mediated ROS genesis in AGEs-induced EPCs.	[27]

Endothelial dysfunction	Oxidative stress, HMGB-1, RAGE, ERK, NF-*κ*B	HMGB-1 induced endothelial dysfunction via oxidative stress and RAGE/ERK/NF-*κ*B pathway.	[34]

Endothelial dysfunction	HMGB-1, RAGE, MMP9	Serum HMGB-1 may reflect endothelial dysfunction developing in DM.	[22]

Cardiomyocyte apoptosis	HMGB-1, ERK, Ets-1, caspase-3, Bax/Bcl-2	HMGB-1 mediated hyperglycaemia-induced cardiomyocyte apoptosis.	[28]

CAD: coronary artery disease; T2DM: type 2 diabetes mellitus; AMI: acute myocardial infarction; DM: diabetes mellitus; DCM: diabetic cardiomyopathy; TGF-1beta: transforming growth factor-1beta; MMP2: matrix metalloproteinase-2; MMP9: matrix metalloproteinase-9; ERK: extracellular signal-regulated kinase; JNK: c-Jun N-terminal kinase; HG: high glucose; RAGE: receptor for advanced glycation end product; VCAM: vascular cell adhesion molecule; NF-*κ*B: nuclear factor-*κ*B; I/R: ischemia-reperfusion; TNF-*α*: tumor necrosis factor-*α*; IL-6: interleukin-6; EPC: endothelial progenitor cell; ROS: reactive oxygen species; AGEs: advanced glycation end products; HUVEC: human umbilical vein endothelial cell; Bax/Bcl-2: ratio of Bcl-2-associated X protein to B-cell lymphoma/leukemia-2; Ets-1: E26 transformation-specific sequence-1; Ref.: references.

**Table 2:** HMGB-1: evidence in DN (diabetic nephropathy).

Involved signal molecule(s)	Notes	Ref.
HMGB-1,TLR4	Strong HMGB-1 staining was detected in proximal and distal tubules of DN biopsies.	[48]

HMGB-1, TLR2, TLR4, MyD88, NF-*κ*B	HMGB-1 and TLRs were upregulated in kidneys of diabetic rats, which were associated with increased renal expression of MyD88 and MCP-1 and activation of NF-*κ*B.	[49]

HMGB-1, TLR4	Upregulated expression of HMGB-1 and TLR4 in early diabetic kidney mice.	[50]

HMGB-1, TLR4, MyD88, SyK, NF-*κ*B, TGF-1beta	ROS-dependent HMGB-1 expression led to Syk activation via binding to TLR4.	[11]

HMGB-1, NF-*κ*B	HMGB-1 mediated the D-glucose-induced proinflammatory cytokines in mesangial cells.	[51]

HMGB-1, RAGE, TLR4, CTGF, TGF-beta	HMGB-1 enhanced AGE-induced expression of CTGF and TGF-beta via RAGE and TLR4-dependent signaling.	[35]

HMGB-1, NF-*κ*B, TNF-*α*, IL-6	HMGB-1 was involved in high-glucose-induced vascular smooth muscle cell proliferation and migration.	[52]

DN: diabetic nephropathy; TLR: Toll-like receptor; MyD88: myeloid differentiation factor-88; NF-*κ*B: nuclear factor-*κ*B; SyK: spleen tyrosine kinase; TGF-beta: transforming growth factor-beta; RAGE: receptor for advanced glycation end products; CTGF: connective tissue growth factor; AGE: advanced glycation end product; Ref.: references.

**Table 3:** HMGB-1 in DR (diabetic retinopathy).

Pathological phenomenon(s)	Related signal molecule(s)	Notes	Ref.
Angiogenesis	HMGB-1, VEGF, sVE-cadherin, sEng	HMGB-1, VEGF, sVE-cadherin, and sEng levels were higher in PDR patients than in nondiabetics. HMGB-1 was positively correlated with sVE-cadherin.	[53]

Angiogenesis	HMGB-1, RAGE, VEGF, CXCL12/CXCR4, HIF-1*α*, early growth response-1, tyrosine kinase-2	HMGB-1 induced upregulation of CXCL12/CXCR4, HIF-1*α*, early growth response-1, and tyrosine kinase-2 in diabetic retinas. HMGB-1 induced VEGF and VEGFR2 expression in HRMEC.	[54]

Angiogenesis and fibrosis	HMGB-1, OPN, CTGF	Upregulated HMGB-1 level in PDR and PVR patients compared with quiescent RD patients.	[55]

Neovascularization	HMGB-1, VEGF-A	HMGB-1 mediated AGE-induced VEGF-A production in RGC-5 cells.	[56]

Inflammation, neovascularization, and hemorrhage	HMGB-1, MCP-1, sICAM-1	HMGB-1 was related to neovascularization and hemorrhage in PDR patients. HMGB-1 expression was upregulated in the retinas of diabetic mice.	[57]

Inflammation and disrupted retinal vascular barrier	HMGB-1, RAGE, ERK, NF-*κ*B, ICAM-1	Increased expression of HMGB-1, RAGE, ERK, and NF-*κ*B in diabetic retinas. HMGB-1 reduced transendothelial electrical resistance of bovine retinal endothelial cells. HMGB-1 induced upregulation of ICAM-1, sICAM-1, HMGB-1, RAGE, ERK, and NF-*κ*B and increased retinal vascular permeability.	[58]

Inflammation	HMGB-1, RAGE, NF-*κ*B	Cytoplasmic translocation of HMGB-1 in diabetes and high glucose in retinal pericytes.	[59]

Inflammation	HMGB-1, NF-*κ*B, TNF-*α*, VEGF	HMGB-1, receptors for HMGB-1, NF-*κ*B, TNF-*α*, and VEGF were upregulated in diabetic retinas and HG-induced ARPE-19 cells. HMGB-1 blockage alleviated HG-induced expression of NF-*κ*B and VEGF in ARPE-19 cells.	[29]

Apoptosis	HMGB-1, TLR4, NF-*κ*B	Inhibition of HMGB-1 decreased expressions of TLR4 and NF-*κ*B in high-glucose-induced RGC-5 cells.	[60]

Apoptosis	HMGB-1, NADPH oxidase, IL-1beta, Nox2, PARP-1, caspase-3	HMGB-1 and oxidative stress levels in vitreous fluid from PDR patients were higher than in controls. HMGB-1 was positively associated with oxidative stress level. HMGB-1 induced IL-1beta, ROS, Nox2, PARP-1, and cleaved caspase-3 expressions in HRMEC and in the retinas of rats.	[61]

Apoptosis	HMGB-1, RAGE	HMGB-1/RAGE expressions as well as apoptosis cells in diabetic rat retina were higher than in controls.	[62]

Neurodegeneration	HMGB-1, BDNF, TBARS, caspase-3, sRAGE, sICAM-1	Decreased serum BDNF and increased serum HMGB-1, sRAGE, sICAM-1, and TBARS in PDR patients. HMGB-1 was negatively correlated with BDNF. HMGB-1 induced upregulation of TBARS and cleaved caspase-3 and downregulated expression of BDNF and synaptophysin in rat retinas.	[63]

VEGF: vascular endothelial growth factor; sVE-cadherin: soluble vascular endothelial-cadherin; sEng: soluble endoglin; PVR: proliferative vitreoretinopathy; OPN: osteopontin; CTGF: connective tissue growth factor; RD: rhegmatogenous retinal detachment with no PVR; MCP-1: chemoattractant protein-1; sICAM-1: soluble intercellular adhesion molecule-1; BDNF: brain-derived neurotrophic factor; TBARS: thiobarbituric acid reactive substances; sRAGE: soluble receptor for advanced glycation end products; HRMEC: human retinal microvascular endothelial cell; IL-1beta: interleukin-1beta; Nox2: NADPH oxidase-2; PARP-1: poly(ADP-ribose) polymerase-1; NF-*κ*B: nuclear factor-*κ*B; ERK: extracellular signal-regulated kinase; HIF-*α*: hypoxia-inducible factor-1*α*; VEGFR2: vascular endothelial growth factor receptor-2; AGE: advanced glycation end product; RGC-5: retinal ganglion cell line 5; Ref.: references.
